# Early term effect of ureterorenoscopy (URS) on the Kidney: research measuring NGAL, KIM-1, FABP and CYS C levels in urine

**DOI:** 10.1590/S1677-5538.IBJU.2016.0638

**Published:** 2017

**Authors:** Erdal Benli, Sema Nur Ayyildiz, Selma Cirrik, Tevfik Noyan, Ali Ayyildiz, Abdullah Cirakoglu

**Affiliations:** 1 Department of Urology, Faculty of Medicine, Ordu University, Ordu, Turkey; 2 Department of Biochemistry, Faculty of Medicine, Ordu University, Ordu, Turkey; 3 Department of Physiology, Faculty of Medicine, Ordu University, Ordu, Turkey; 4 Department of Urology, Research and Training Hospital, Ankara, Turkey

**Keywords:** Acute Kidney Injury, Kidney, Lithotripsy

## Abstract

**Aim:**

URS is a very commonly used procedure for treatment of ureter stones. Increased hydrostatic pressure in the collecting system linked to fluids used during the procedure may cause harmful effects on the kidney. The aim of this study is to determine whether the URS procedure has a negative effect on the kidney by investigating NGAL, KIM-1, FABP and Cys C levels in urine.

**Material and Methods:**

This study included 30 patients undergoing ureterorenoscopy (URS) for ureter stones. Urine samples were collected 5 times; before the URS procedure (control) and at 1, 3, 5 and 12 hours following the procedure. NGAL, KIM-1, FBAP and Cys C levels were measured in urine and compared with the control values.

**Results:**

The NGAL levels in urine before the procedure and at 1, 3, 5 and 12 hours after the procedure were 34.59±35.34; 62.72±142.32; 47.15±104.48; 45.23±163.16 and 44.99±60.79ng/mL, respectively (p=0.001). Similarly, the urinary KIM-1, FABP and Cys C levels were found to increase compared to control values; however this increase did not reach statistical significance (p >0.05).

**Conclusions:**

After the URS procedure, there were important changes in NGAL, FABP, KIM-1 and Cys C levels. These changes reached statistical significance for NGAL, but did not reach significance for the other parameters. In conclusion, the URS procedure significantly affects the kidney; however, this effect disappears over time.

## INTRODUCTION

URS, used as an alternative to open surgery and ESWL for stone treatment, has revolutionized treatment of stones located in the upper urinary system. It is reported to be a reliable and effective procedure even for complex cases like solitary kidney, pregnancy and obesity. However, the procedure has potential for serious complications like ureter injury and kidney loss (1, 2).

The majority of complications reported for URS are related to traumatic injury caused by the direct effect of surgical tools on tissue. The effect of the procedure on the kidney has not been sufficiently revealed. Processes related directly to stone disease and to treatment may affect renal functions. There are limited studies on this topic and data are mostly related to the late period. The effect of the procedure on renal functions in the early period is not fully known. The small amount of data related to renal injury in the post-op period may be due to a lack of focus on the topic or problems with diagnosis. It is well known that markers like urea and creatinine used to monitor renal functions are not sufficiently reliable to determine renal injury in the early period. Another reason may be that patients are discharged from hospital before classic symptoms of renal injury occur. When data is obtained from patients requiring long post-op monitoring in hospital, renal injury appears to be more common than realized. A study on this topic found that renal injury occurred in 40% of patients after cardiac surgery (3).

Recently, some biomarkers have been suggested with the ability to better identify renal damage in the early period compared with traditional markers. The focus has mainly been on markers like neutrophil gelatinase-associated lipocalin (NGAL), cystatin C (Cys-C), kidney injury molecule-1 (KIM-1) and liver-type fatty acids binding protein (L-FABP) and they are reported to identify renal injury earlier compared to creatinine (4).

To the best of our knowledge in the literature to date, the effect of the URS procedure on the kidney has not been investigated using the new biomarkers NGAL, KIM-1, FABP and Cys-C. The aim of this study is to determine whether the URS procedure commonly used for stone treatment has a negative effect on the kidney by measuring these new biomarkers in urine.

## MATERIAL AND METHODS

### 

#### Study Design

This study was approved by the local ethics committee of Samsun University (2014/556). The study included 30 patients submitted to URS procedure due to ureter stones in 2015 and 2016. Before surgery direct urinary system graphy, urinary ultrasound, intravenous pyelography, urine tests, urea, creatinine and other necessary tests were completed. The exclusion criteria for the study were age under 18 years, use of permanent catheter, urinary tract infection, muscular diseases, use of cytotoxic medication, renal failure, and diseases that affect marker levels in urine or serum like protein leak. Additionally, cases with non-functioning kidney occurring due to the stone were excluded from the study. The patients signed a consent form after being informed about the procedure.

The surgical procedure was completed under general anesthesia in the lithotomy position. During the procedure, a 10F semi-rigid ureteroscope (Storz, Germany) and laser lithotriptor (Stonelight laser) were used. Urine samples were taken before surgery with a 10F nelathon catheter and after surgery with the aid of a 14F Foley catheter inserted in the bladder. Urine samples were obtained from patients 5 times; immediately before the URS procedure (baseline) and 1, 3, 5 and 12 hours after the procedure. Samples were stored at − 80°C until laboratory study.

## BIOCHEMICAL STUDY

### 

#### Urinary Cystatin C measurement

Measurements were completed with Bio-vendor brand Human Cystatin C ELISA kits (Lot No: E15-001, Cat No: RD191009100) using the Sandwich-ELISA method. Urine samples were diluted 1/20 and read at 450nm wavelength with an ELISA reader (BioTek, ELx800).

#### Urinary FABP measurement

FABP measurements were completed with Elabscience brand Human FABP3 ELISA kits (Lot No: AK0015APR21031 Catalog No: E-EL-H1431) using the Sandwich-ELISA method. Results were read at 450nm wavelength with an ELISA reader (BioTek, ELx800).

#### Urinary NGAL measurement

Measurements used Boster Immunoleader brand Human Lipocalin-2/NGAL ELISA kits (Lot No: 5031059708 Catalog No: EK0853) with the Sandwich-ELISA method. Samples were diluted 1/10 and results were read at 450nm wavelength with an ELISA reader (BioTek, ELx800).

#### Urinary KIM-1 measurement

Measurements used the Sandwich-ELISA method with Boster Immunoleader brand Human KIM1 ELISA kits (Lot No: 5299125708 Catalog No: EK0883). Results were read at 450nm wavelength with an ELISA reader (BioTek, ELx800).

#### Urinary Creatinine measurement

Abbott brand trade creatinine kits (Creatinine Lot No: 78067UN14) were used in an Abbott Architect C8000 autoanalyzer at the Ministry of Health Ordu University Education and Research Hospital Biochemistry Laboratory.

Markers measured in urine were normalized according to 24-hour urine volume and creatinine value.

### Statistical analysis

Data obtained from repeated measurements in the study were assessed with variance analysis. The results are given as sample size, mean and standard deviation (SD). All statistical analyses were completed with “SPSS for Windows Version 20.0” program. Data with p value <0.05 were accepted as significant.

## RESULTS

The mean age of patients was identified as 52.7±12.7 (28-78 years) (mean±SD) (min-max). According to sex, age distribution for females and males were identified as 53.1±13.8 years (31-78) and 51.6±12.4 years (28-72), respectively (p=0.707). Mean stone size was 9.7±1.8mm and operation duration was 24.8±9.3 minutes. In terms of stone localization, 15 patients (50%) had lower ureter, 11 patients (36.7%) had middle ureter and 4 patients (13.3%) had upper ureter stones. No serious complication was encountered after surgery. No patient had a double-J (DJ) stent inserted. Creatinine values in serum before the procedure and 10 days after the procedure were 0.81±0.15 and 0.89±0.31, respectively, and the variation was not statistically significant (p=0.117).

The NGAL values measured in urine were identified as 34.59±35.34 for baseline, 62.72±142.35 for 1st hour, 47.15±104.48 for 3rd hour, 45.23±163.16 for 5th hour and 44.99±60.79ng/dL for the 12th hour. The variation in NGAL values was statistically significant (p=0.001). Additionally, there were differences in the variation of this parameter within the group (p<0.05) (Table-1).

**Table 1:** Time-linked variation in NGAL (ng/dL) measured in urine.

Parameter	Mean	Std.Dev	P-value	Linear	Quadratic	Cubic	Order 4
NGAL-0	34.59^a^	35.34					
NGAL-1	62.72	142.32					
NGAL-3	47.15	104.48	0.001	0.010	0.062	0.541	0.157
NGAL-5	45.23^a^	163.16					
NGAL-12	44.99	60.79					

a significant difference within the group.

The variation in FABP (pg/mL) during the URS procedure was similar; 128.59±104.48, 214.50±251.57, 192.25±163.16, 192.76±142.32 and 164.76±218.90, respectively. There was an increase in FABP value during the procedure compared to the baseline value. However, this increase did not reach statistical significance (p=0.292). Additionally, there were no differences in terms of this parameter within the group (p>0.05) (Table-2).

**Table 2:** Time-linked variation in FABP (pg/mL) measured in urine.

FABP	Mean	Std. Dev	p-value
Control	128.59	104.48	
1st hour	214.50	251.57	
3rd hour	192.25	163.16	0.292
5th hour	192.25	142.32	
12th hour	164.76	218.90	

No differences between the groups

The variation in KIM-1 (ng/mL) was as follows; 2.06±3.71, 3.62±6.43, 2.49±2.49, 2.28±2.75 and 2.26±2.40. Though KIM-1 levels increased during the procedure compared to baseline values, this variation was not significant (p=0.707). Additionally, there were no differences within the group (p >0.05) (Table-3).

**Table 3:** Time-linked variation in KIM-1 (ng/mL) measured in urine.

KIM 1	Mean	Std. Dev	P -value
Control	2.0662	3.71348	
1st hour	3.6234	6.43728	
3rd hour	2.4924	2.49448	0.707
5th hour	2.2866	2.75137	
12th hour	2.2610	2.40135	

No differences between the groups

The variation in Cys-C (ng/mL) levels occurred as follows 27.38±25.20, 55.70±81.53, 45.70±71.17, 37.60±46.37 and 33.49±27.60. Though the Cys-C levels increased compared to baseline values, this increase did not reach statistical significance (p=0.095). There were no differences in terms of this parameter within the group (p <0.05) (Table-3). The creatinine values measured in serum before and after the procedure (mg/dL) were 0.89±0.31 and 0.81±0.15, respectively (p=0.117) (Table-4).

**Table 4:** Variation in Cys-C (ng/mL) measured in urine.

Cys C	Mean	Std. Dev	P -value
Control	27.38	25.20	
1st hour	55.70	81.53	
3rd hour	45.70	71.17	0.095
5th hour	37.60	46.37	
12th hour	33.49	27.60	

No differences between the groups

## DISCUSSION

This study observed that the URS procedure affected kidney functions with increases measured in urinary NGAL, Cys-C, KIM-1 and FABP levels in the early period. The variations in biomarkers after the procedure only reached statistical significance for NGAL.

The first imaging of the upper urinary system in 1912 brought about significant changes in surgical procedures related to the upper urinary system. The advances in endoscopic systems have brought URS to the forefront of treatment for ureter and kidney stones. Significant advantages of these procedures include effectiveness and reliability of many procedures performed on the upper urinary system in addition to providing rapid post-op recovery (5). The majority of complications encountered during the URS procedure are insignificant and can be treated with simple approaches. A study investigating complications developing after URS reported the general complication rate after URS was 5.9% for 2436 URS cases. Among the most commonly encountered intraoperative problems are mucosal edema, mucosal injury, false passage, ureteral perforation and ureteral avulsion (6).

The majority of reported complications related to URS are due to traumatic effects of the tools used. The correlation of this procedure to acute kidney injury (AKI) is not fully known. It is known that problems affecting kidney functions like obstructive uropathy, surgical procedures and urinary sepsis are commonly encountered in this patient group. As a result, urologic patients may be considered a risk group in terms of renal functions. As the URS procedure is frequently reliable, post-op renal functions are not monitored. Patients are discharged from hospital on the same day or following day post-op and generally renal functions are not monitored in this period. Additionally, markers like urea and creatinine examined in this early period may be misleading in terms of showing renal functions in the late period. In conclusion, the effect of the URS procedure on renal tissue in the early period has not been fully researched to date. This study was designed to determine the effect of the URS procedure on early period renal functions. One of the rare studies on this topic by Caddeo et al. investigated the incidence of AKI developing after the URS procedure. The results of this study identified that AKI developed in nearly 3.6% of cases (7). However, in this study renal functions were based on serum creatinine evaluated in the late period and no information was given about the early period. Another study by Ghosh et al. assessed the effect of the URS procedure in a solitary kidney. The authors reported that renal functions were not affected by the procedure (8). This study assessed renal functions in the 3rd month post-op and did not study results related to the early period.

In these studies, mostly late stage kidney functions were investigated and no information was given about the early period. As observed in our study results, renal functions are affected in the early period. In the literature, renal functions in the early function after URS are not known due to the scarcity of studies.

The surgical tools and the high-pressure fluid used during the URS procedure are considered to possibly affect renal functions. A study on this topic by Schwalb et al. investigated the effect of fluid pressure in an animal model. This study identified morphologic changes in renal tissue in the group with high pressure fluid used compared to the baseline group in both the early and late period. The authors reported the irrigation fluid used during the procedure had a damaging effect on the kidney (9). However, when the literature is examined most data relating to AKI developing after surgery comes from cardiac surgery studies. The reason for this may be that after cardiopulmonary surgery patients are frequently monitored in surgical intensive care units (ICU) for several days. Thus, there is sufficient time for markers of renal injury in serum to change. The result of this close monitoring is that some studies report about 40% development of AKI post-op (3). One of the rare studies related to urologic surgery by Caddeo et al. reported the incidence of AKI in patients after urologic surgery was 0.7-43% (7). As shown by this study, renal injury after surgery is commonly encountered. However, due to problems with diagnosis of this disease, it may not be noticed. As a result, as interest in this topic increases, it is expected that as with other surgical branches, urology clinics will have an increase in the incidence of renal injury. Diagnosis of AKI, with high morbidity and mortality rates, in the early period is important in order to take necessary precautions, to avoid factors affecting kidney functions and for replacement treatment. Studies have reported that AKI lengthens hospital stays, increases labor losses and treatment costs and causes mortal complications (10).

As with many surgeries, it may be difficult to identify renal injury developing after the URS procedure. The main reasons for this are that patients are discharged on the same day or on the following day post-op and renal functions are rarely checked in this period. Another reason is the lack of a reliable imaging method or marker to show renal injury in the early period. Markers commonly used for AKI diagnosis like urea and creatinine are reported to be insufficient to identify kidney damage in the early period and to predict severity and results. Additionally, it is known that markers like urea and creatinine measured in serum are affected by a variety of factors like age, sex, fluid and protein intake and muscle mass (4, 11). In conclusion, it may be misleading to assess renal injury occurring in the early period after URS with serum urea and creatinine values. As renal functions are not seriously affected at this point, renal injury may not be noticed by the patient.

Studies in recent times have proposed some newly-emerging markers that are superior to creatinine to identify renal injury in the early period. The most noteworthy of these markers are NGAL, Cys-C, KIM-1 and FABP (12).

NGAL is a small protein from the lipocalin family with 25kDA weight. Recent studies have reported that NGAL begins to increase in the early period of renal injury. A study on this topic reported that NGAL may be effective in terms of identification in the early period after contrast nephropathy (13). Another study compared NGAL with serum creatinine values for identification of early stage renal injury. The authors examined serum creatinine values to diagnose renal injury and reported this was 1-3 days delayed compared to NGAL (14). Another study investigated urinary NGAL levels to identify diabetic nephropathy in the early period. The results of this study reported that it may be a beneficial biomarker to identify renal injury in the early period (15). The same study reported a correlation between severity of renal injury and NGAL levels.

The power of NGAL to identify renal injury in the early period after surgery was investigated by Mishra et al. The authors reported that NGAL can identify renal injury within 2 hours post-op (16). In our study the urinary NGAL levels in the first hours after the URS procedure were significantly increased compared to the baseline value. Later, the levels of this marker reduced in a time-linked fashion. In conclusion, the URS procedure affects renal functions in the early post-op period. This situation should be considered for critical patients in terms of renal functions.

Cys-C is a cystein proteinase enzyme inhibitor. As it has low molecular weight and does not bind to proteins, it is freely filtered by glomerules. Under normal conditions it is reabsorbed by the proximal tubules and has low levels in urine. Any factor affecting the proximal tubule structure affects reabsorption of Cys-C. It is considered a sensitive marker of renal tubular injury (17). The study by Hall et al. reported that it was a beneficial maker to identify renal injury developing in the early period in patients in the ICU after surgery (18). For early identification of renal injury, Pirgakis et al. compared serum creatinine levels with Cys-C levels in surgical patients. The authors reported that the Cys-C value measured in the 6th hour post-op was a strong predictor of late period renal function (19). Another study by Liangos et al. reported that Cys-C increased in urine in the early period in ICU patients developing renal injury (20). The power of Cys-C for early identification of renal injury developing after urologic surgery has been investigated in several studies. One of these studies compared early identification of renal injury after partial/radical nephrectomy with serum creatinine levels on the 1st day post-op. The authors reported Cys-C performance was superior to creatinine to determine renal injury developing in the late period (21). Koyner et al. compared the performance of Cys-C, NGAL, and KIM-1 to identify renal injury developing in the early period after cardiac surgery. This study suggested that Cys-C provided the best performance in terms of identifying renal injury in the early period (22). The authors also reported a correlation between this marker and mortality. The results of our study identified an increase in urinary Cys-C levels in the early post-op period compared to baseline values. This increase reduced over time; however, it did not reach statistical significance.

Studies related to KIM-1 have reported it may be a beneficial marker to identify renal injury in the early period (4). A study by Han et al. investigated the performance of KIM-1 to identify renal damage developing post-op in the early period. This study showed that from the 3rd hour after injury urinary levels began to increase. The authors reported that KIM-1 was a beneficial marker to identify AKI in the early period (23). Liangos et al. reported that urinary KIM-1 levels were a beneficial marker to determine prognosis of patients in the ICU developing AKI (24). Another study by Song et al. investigated the correlation between KIM-1 levels and tissue injury occurring in renal tissue in renal transplant patients developing tissue rejection. The researchers reported a strong correlation between KIM-1 levels and tubular cell and tissue injury shown with biopsy in patients developing rejection. They reported that this marker began to increase before tissue damage developed or before traditional serum markers began to increase (25). In our study the KIM-1 levels measured in urine increased especially in the first hours after the URS procedure compared to baseline values. Though this increase was not statistically significant, the damaging effect of the URS procedure on the kidney was observed in the increase in urinary KIM-1 levels.

Under normal conditions there are trace amounts of FABP in urine, with the amount increasing when renal injury develops. A study by Matsui et al. compared FABP with creatinine in terms of determining AKI in the early period. The results indicate FABP identified renal injury earlier compared to creatinine (26). Yamamoto et al. investigated the performance of FABP to determine early stage renal injury in both animal models and kidney transplant patients. The authors reported that FABP in urine was a beneficial biomarker to predict acute ischemic injury developing after ischemia-reperfusion injury (27). This opinion was later supported by Matsui et al. (28). Another study by Manabe et al. showed that urinary FABP levels were good indicators to predict AKI developing linked to contrast nephropathy (29). Another study investigated the correlation between urinary FABP, KIM-1, VENAG and NGAL levels and prognosis for terminal renal injury. This study reported that FABP and NGAL were effective to predict results of terminal renal injury (30). In our study the urinary FABP values increased in the early post-op period after the URS procedure compared to baseline values. However, this variation did not reach statistical significance. In accordance with our results, in the literature some studies have not shown positive results for FABP.

This study investigated the use of new markers reported to show renal injury in the early period to determine the effect of the URS procedure on the kidney. To the best of our knowledge this study is the first on this topic in the literature. This study showed that kidney functions were affected in the early period with increases occurring in the markers in urine. The results of the study were that levels of all markers increased in urine in the first hours (especially 1st and 3rd hours) and later reduced in a time-linked fashion. The variation in marker levels only reached significance for NGAL. From these results, it is understood that the URS procedure affects kidney functions in the early period. It is very difficult to explain the cause of this injury because there are insufficient studies on the effect of the URS procedure on the kidney in the early period.

A cause of this injury may be reduced renal blood perfusion, though this was not investigated in our study. The high intra-pelvic pressure caused by the fluid used during the procedure may cause a reduction in renal blood perfusion. Another cause may be medications used during anesthesia with contributions from hypotensive attacks occurring during the procedure and many other unknown reasons. There is a need for detailed studies on this topic.

Our study is the first study to evaluate the results of the URS procedure in the early period using new markers in urine. As a result, there is no study in the literature that we can use to directly compare our results.

There are some limitations to this study. Among these are the low number of cases and the fact that the study was completed at a single center. Also, the lack of knowledge about renal blood perfusion during the procedure is another significant limitation. However, as this is the first study on this topic and the results are noteworthy we consider this to be a significant study.

## CONCLUSIONS

In conclusion, in our study the URS procedure had significant effects on the kidney with an increase in NGAL, FABP, KIM-1 and Cys C levels examined in urine. However, these variations only reached statistical significance for NGAL. According to our idea, the URS procedure is not the harmless procedure it is generally accepted to be. As a result, kidney functions should be closely monitored, especially for patients with borderline kidney functions. Classic markers like urea and creatinine are not sufficiently reliable to show damage in the early period. As shown by our results, after more comprehensive studies new markers may be beneficial in identifying renal damage in the early period and in terms of taking necessary precautions.
